# Jaw Function in *Smilodon fatalis*: A Reevaluation of the Canine Shear-Bite and a Proposal for a New Forelimb-Powered Class 1 Lever Model

**DOI:** 10.1371/journal.pone.0107456

**Published:** 2014-10-01

**Authors:** Jeffrey G. Brown

**Affiliations:** Anatomic and Clinical Pathology, Independent Researcher, New York, New York, United States of America; Université de Poitiers, France

## Abstract

The jaw function of *Smilodon fatalis* has long been a source of debate. Although modern-day lions subdue large prey through the use of a suffocating throat bite, the dramatically elongated maxillary canines of *S. fatalis* suggest an alternative bite mechanism. The current literature favors a “canine shear-bite,” in which the depression of the cranium by the ventral neck flexors assists the mandibular adductors in closing the jaws. Although the model makes intuitive sense and appears to be supported by scientific data, the mechanical feasibility of “neck-powered” biting has not been experimentally demonstrated. In the present study, the computer-assisted manipulation of digitized images of a high-quality replica of an *S. fatalis* neck and skull shows that a rotation of the cranium by the ventral neck flexors will not result in jaw closure. Instead, the cranium and mandible rotate ventrally together (at the atlantooccipital joint), and the jaws remain in an open configuration. The only manner by which rotation of the cranium can simultaneously result in jaw closure is by an anterior rotation at the temporomandibular joint. Based on this finding, the author proposes a new Class 1 lever mechanism for *S. fatalis* jaw function. In this model, the mandible is immobilized against the neck of the prey and a dorsally directed force from the extension of the forelimbs rotates the cranium anteriorly at the temporomandibular joint. The maxillary canines pierce the prey’s neck and assist in clamping the ventral neck structures. The model is based on a maximum gape angle of approximately 90° and incorporates a secondary virtual point of rotation located slightly anteroventral to the temporomandibular joint. The Class 1 Lever Model is mechanically feasible, consistent with current data on *S. fatalis* anatomy and ecology, and may provide a basis for similar studies on other fossil taxa.

## Introduction

The late Pleistocene sabertooth cat, *Smilodon fatalis,* with its powerful forelimbs and knife-like maxillary canines, is one of the most visually striking and enigmatic of the extinct Ice Age mammals. This large predatory cat was similar in size to the modern-day lion but had a more powerful build [1], [2], and it is thought to have hunted large, thick-skinned herbivores, including bison, camels and horses [3]–[9]. Although *S. fatalis* was a successful predator that flourished during its epoch [10], [11], the manner in which it killed its prey remains poorly understood. The convergent evolution of similar “sabertooth complexes” [10] within multiple independent carnivore lineages (non-mammalian cynodonts, creodonts, nimravids, barbourofelidae, machairodontine placentals and sparassodont metatherians [12]) suggests an important selective advantage for the sabertooth bite mechanism [10], [12], [13].

There is a mechanical trade-off in carnivore evolution between an enlarged gape and a forceful bite [3], [14]–[17]. As the maximum gape increases in size, a subsequent loss in mechanical advantage results in a less forceful bite [13], [18]. Given the extended length of the *S. fatalis* maxillary canines and the need for a substantially enlarged gape, it seems unlikely that the mandibular adductors could generate sufficient force for the cat to use a suffocating throat bite [3], [12], [19]. As a result, paleontologists have long argued that the *S. fatalis* maxillary canines functioned in a unique manner, perhaps by stabbing or slashing the prey [4]–[6], [20]. In the most widely accepted model, known as the “canine shear-bite,” the cat augments the jaw-closing force of its mandibular adductors with a ventral nodding motion of the head [20]–[22]. The strike is directed at the prey’s ventral neck, where there is an increased chance of major vascular injury [11], [13], [23].

Despite its widespread acceptance in the paleontology community [8], [10], [12], [19], [20], [22], [24]–[28] and its seeming support from anatomical [20], [22], [25] and comparative [12], [21], [29]–[32] studies, the mechanical feasibility of “neck-powered” biting [21] has not been experimentally demonstrated. More specifically, can the forces generated by the ventral neck flexors contribute to the forces generated by the mandibular adductors and thereby increase the force for closing the jaws?

## Materials and Methods

An anatomical model for studying *S. fatalis* bite mechanics was assembled using a high-quality, life-size, polyurethane replica of an adult *S. fatalis* cranium, mandible and neck (cervical vertebrae 1–7). This replica was produced from a composite skeleton cast of *S. fatalis* from Rancho La Brea (Los Angeles, CA) and manufactured by Bone Clones (Canoga Park, CA, USA) (www.boneclones.com) under license from the Natural History Museum of Los Angeles County. The author purchased the replica and did not have access to museum collections. The cranium and mandible represented associated specimens from Rancho La Brea Pit 67. The LACMHC catalog numbers are as follows: LACMHC 2001-249 (skull), LACMHC 2002-L&R-250 (mandible), LACMHC 106639 (I2, right), LACMHC N-1123 (C1), LACMHC N-1908 (C2), LACMHC N-2470 (C3), LACMHC N-2824 (C4), LACMHC N-3451 (C5), LACMHC N-4202 (C6) and LACMHC N-4707 (C7). The Bone Clones catalog numbers are as follows: BC-018A (cranium and mandible) and SC-018-08 (cervical vertebrae 1–7).

**Figure 1:**
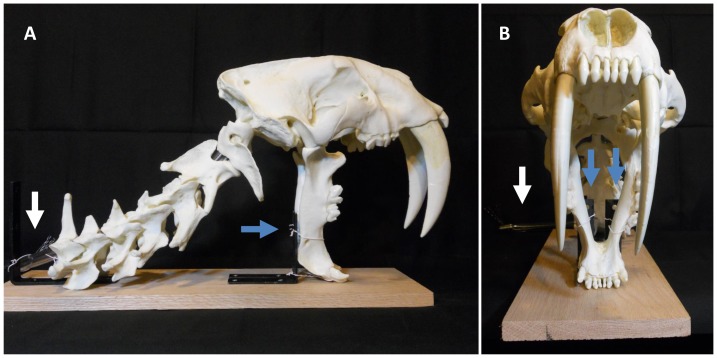
Unmodified digitized image of the anatomical model used for studying bite mechanics in *S. fatalis*. (A) Lateral view. (B) Frontal view. The mandible has been fixed in a vertical position (simulating its vertical position against the neck of the prey) using a wooden base, metal brackets and wire. The cranium and vertebrae have been articulated using flexible vinyl tubing. Blue arrows: metal brackets; white arrow: vinyl tubing.

The cranium and cervical vertebrae were articulated using flexible vinyl tubing (outer diameter ¾ in, inner diameter ½ in), which was secured at each end with metal hardware. The mandible was fixed in a vertical position (simulating its vertical position against the neck of the prey) using a wooden base, metal brackets and wire [Figs. 1A, B]. Lateral-view photographs were taken using a Nikon CoolPix S3000 camera. Simple editing was performed using Adobe Photoshop image software (i.e., the background was removed).

For the evaluation of the canine shear-bite, the forces contributed by the ventral neck flexors were modeled geometrically as a ventral rotation of the cranium at the atlantooccipital joint (AOJ) [20]. Because the mandible in the canine shear-bite is hypothesized to remain stationary, the forces contributed by the mandibular adductors were modeled geometrically as an anterior rotation of the cranium at the temporomandibular joint (TMJ) [20]. The ability of the forces generated by the ventral neck flexors to contribute to the forces generated by the mandibular adductors was assessed visually by the ability of a rotation of the cranium at the AOJ to result in a rotation of the cranium at the TMJ. The rotation of the cranium at the TMJ was verified by the ability of the TMJ to remain stationary during the rotation.

**Figure 2:**
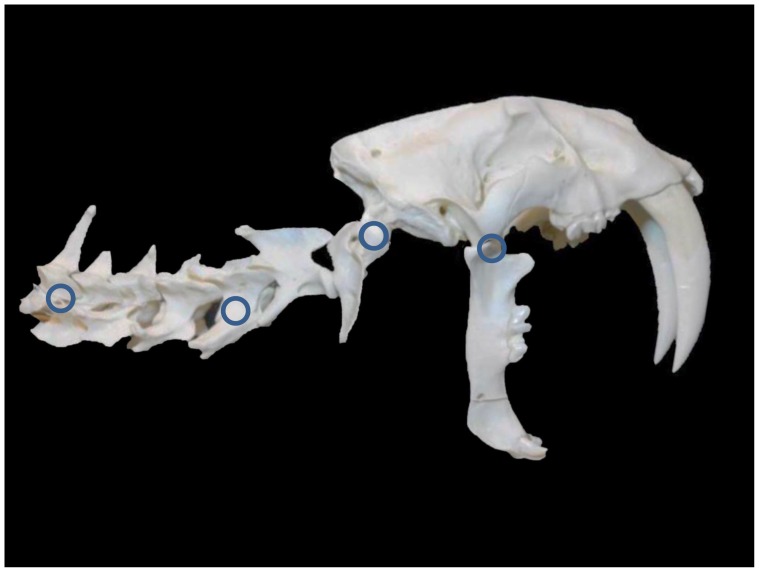
Experimental (modified) digitized image of *S. fatalis* neck and skull. Four hypothesized points of rotation (blue circles) are indicated: the caudal neck, the mid-neck, the atlantooccipital joint and the temporomandibular joint. For the purposes of the experiment, the original image (see Fig. 1A) was digitally manipulated to more fully extend the cranium at the atlantooccipital joint and to position the jaws at a maximum gape of 90°.

**Figure 3:**
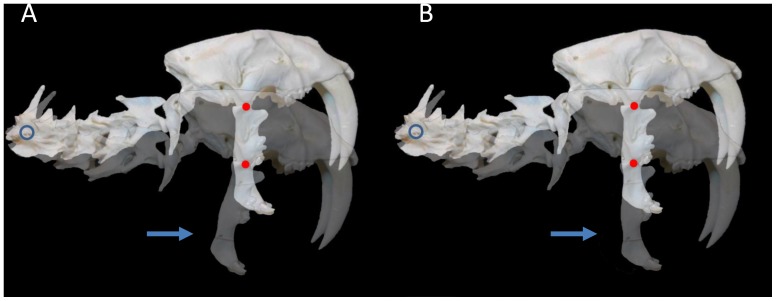
Experimental image of *S. fatalis* neck and skull rotated 15° ventrally at the caudal neck. (A) The mandible is held in a constant position relative to the cranium. (B) The mandible can rotate 15° dorsally to return to a vertical position. Blue circle: point of rotation; red dot: temporomandibular joint; arrow: mandible.

**Figure 4:**
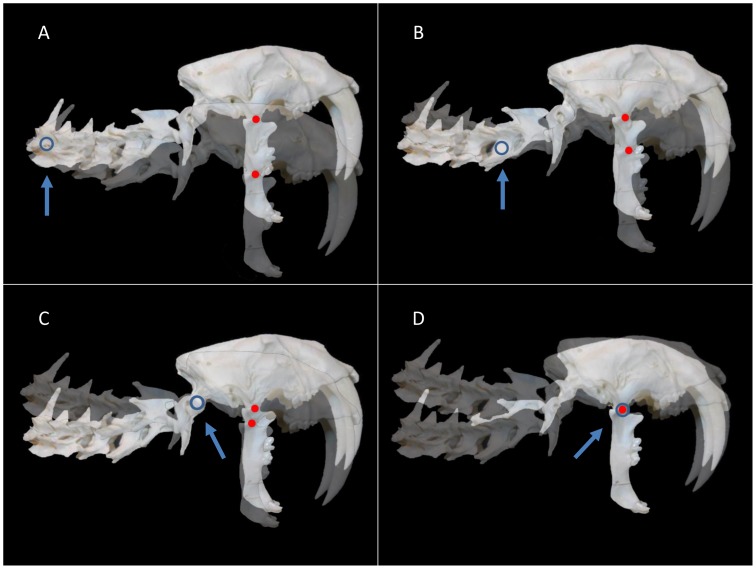
Experimental image of *S. fatalis* neck and skull rotated 15° ventrally at four points. The experimental image has been rotated 15° ventrally at the (A) caudal neck, (B) mid-neck, (C) atlantooccipital joint and (D) temporomandibular joint. The mandible can return to a vertical position. Note that the temporomandibular joint is the only point of rotation for the neck and skull that allows the mandible to remain stationary. Blue circle: point of rotation; red dot: temporomandibular joint. The blue arrow highlights the point of rotation.

For purposes of the experiment, the original image of the *S. fatalis* neck and skull [Fig. 1A] was graphically manipulated to increase the extension of the cranium at the AOJ (extension was increased by 36°) and to position the mandible at a maximum gape angle of 90° [Fig. 2], a value that is in agreement with the current paleontology literature [12], [21]. The maximum gape angle was measured from the TMJ to the incisor tips after Andersson et al. [21]. For purposes of comparison, four points of rotation were chosen: the caudal neck, the mid-neck, the AOJ and the TMJ [Fig. 2]. Adobe Photoshop was used to rotate the experimental image of the neck and skull ventrally at each point, and the original and rotated images were superimposed to assess the movement of the TMJ. An arbitrary arc of 15° was chosen for the arc of rotation. The mandible was first held in a constant position relative to the cranium [Fig. 3A]. Then, the mandible was allowed to rotate freely at the TMJ to return to a vertical position [Fig. 3B]. Fig. 4 (A–D) shows a side-by-side comparison of the four rotations with the mandible returning to a vertical position. A rotation of the neck and skull was also attempted at each of the four points while keeping the mandible stationary. Finally, limited rotations were performed using the *S. fatalis* polyurethane replica to confirm that the results were not an artifact of the computer modeling.

For the Class 1 Lever Model, the author used images of the neck and skull at two hypothesized bite positions: bulldogging (mandible positioned vertically, cranium and neck fully extended) and strike (mandible positioned vertically, cranium rotated anteriorly at the TMJ and neck fully flexed). The bulldogging image was identical to the experimental image used for the evaluation of the canine shear-bite, except that a small gap was introduced between the cranium and mandible to simulate the TMJ articular cartilage (to more accurately represent the anatomical relationships) [12]. To improve the visualization of the strike sequence, all images of *S. fatalis* were reoriented with the neck on the left and the skull on the right.

To better illustrate the canine shear-bite versus Class 1 Lever Model, Adobe Photoshop was used to reconstruct the theoretical models from a photograph of a museum specimen of a complete skeleton of *S. fatalis*. Care was taken to ensure that the anatomical relationships of the reconstructed models remained within the expected range of a normal cat [33]–[35].

As an evolutionary consideration, the mechanical advantage (MA) of the Class 1 Lever Model and the MA of the mandibular bite were compared using the following equation:


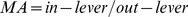
 [36], [37],in which the in-lever is defined as the perpendicular distance from the line of action of the effort force (hereafter referred to as force) to the fulcrum, and the out-lever is defined as the perpendicular distance from the line of action of the resistance force (hereafter referred to as resistance) to the fulcrum [37], [38]. For simplicity, only the temporalis muscle was considered for the MA of the mandibular bite. The out-lever for both mechanisms was modeled (for purposes of illustration) as the distance from the TMJ to the canine tips, although the compressive force of the suffocating throat bite may have been located more posteriorly in the mouth.

## Results

The rotations of the neck and skull at three different points (the caudal neck, the mid-neck and the AOJ) were all accompanied by a ventral translation of the TMJ. Even allowing for the free rotation of the mandible at the TMJ, the mandible was unable to remain stationary. When the mandible was required to remain stationary, the neck and skull were unable to rotate at any of the three points. The only point of rotation for the neck and skull in which the TMJ remained stationary was the TMJ itself.

A hypothetical Class 1 Lever Model for the movement of the *S. fatalis* head and neck was constructed incorporating the bulldogging and strike positions. In this model, the mandible was immobilized in a vertical position and a dorsally directed force from the extension of the forelimbs was used to rotate the cranium and neck anteriorly at the TMJ. A secondary virtual point of rotation (as previously described in the literature [12]) was incorporated into the model. *S. fatalis* anatomical features in support of the Class 1 Lever Model were noted and are described below in the Discussion.

A comparison of the Class 1 Lever Model and mandibular bite showed that both mechanisms share the same fulcrum (the TMJ) and out-lever (perpendicular distance from the line of action of the resistance to the TMJ). Therefore, the ratio of the MA between the two mechanisms is equal to the ratio of their respective in-levers. The increase in the MA of the Class 1 Lever Model compared with the mandibular bite was visually apparent due to the much greater length of its in-lever.

## Discussion

### The Canine Shear-Bite

According to Akersten’s original 1985 description [20], there are two main phases to the canine shear-bite: a “mandibular phase” [Fig. S1 in PowerPoint Slide Show S1], in which there is a dorsal rotation of the mandible through the action of the mandibular adductors, and a “neck-powered phase” [Fig. S2 in PowerPoint Slide Show S1], in which there is a depression of the cranium through the action of the ventral neck flexors. According to Akersten, at the end of the first phase, the mandible is immobilized against the hide of the prey [20]. In the second phase, the neck “drives” the cranium into the stationary mandible [20]. Shearing of the prey’s tissue in the second phase of the bite results from the closure of the jaws with the posterior edge of the maxillary canine moving in a posterior direction relative to the mandibular canine, a motion related to the slightly different centers of rotation of the two teeth [20].

The point of rotation for the canine shear-bite is the AOJ [20], [22], and the depression of the cranium is powered by the ventral neck flexors, primarily the atlantomastoid musculature (i.e., m. obliquus capitis cranialis), the sternomastoid/cleidomastoid and the m. obliquus capitis posterior [20], [22]. Although Akersten originally positioned the strike on the abdomen of the prey, the currently accepted location is the prey’s ventral neck, where the maxillary canines are hypothesized to cut the carotid, resulting in exsanguination of the prey [11], [23], [25], [26], [39].

However, as the results of the present study demonstrate, if the cranium rotates ventrally at the AOJ (or at a point of rotation further caudal on the neck), then it is not possible for the mandible to remain stationary. In this situation, the TMJ (and the mandible) will be translated ventrally, following an arc with a radius equal to the distance from the TMJ to the point of rotation. Alternatively, if the mandible is required to remain stationary (as indicated by Akersten’s description), then the cranium and neck will be fixed at two separate points (the TMJ and the point of rotation), and the system will fail to rotate. Therefore, Akersten’s concept that the immobilization of the mandible against the hide of the prey will somehow allow the ventral neck flexors to close the jaws is mechanically impossible.

The only point of rotation for the cranium that will simultaneously result in jaw closure is an anterior rotation at the TMJ. This observation was most likely recognized by Akersten, whose description and illustration ([20]; Fig. 8) of the canine shear-bite imply an anterior rotation of the cranium at the TMJ (according to Akersten, the mandible is stationary). Nonetheless, the force required to augment the anterior rotation of the cranium at the TMJ is not explained by Akersten’s model.

The fallacy of the canine shear-bite lies in the fact that the forces contributed by the ventral neck flexors and the forces contributed by the mandibular adductors act to rotate the cranium at two different points. Whereas the ventral neck flexors rotate the cranium ventrally at the AOJ [20], with the AOJ stabilized by the neck of the cat [22], [25], the mandibular adductors rotate the cranium anteriorly at the TMJ, which is immobilized by the hide of the prey [20]. As shown by the present study, however, a ventral rotation of the cranium at the AOJ will not contribute to anterior rotation of the cranium at the TMJ (the cranium can rotate at the AOJ or TMJ but cannot simultaneously rotate at two different points). Therefore, if we accept the premise that the mandible is stationary and that the cranium rotates anteriorly at the TMJ (allowing the jaws to close), it becomes necessary to identify another muscular force that can rotate the cranium anteriorly at the TMJ in order to augment the force of the mandibular adductors for closing the jaws at greatly extended gapes.

### Positioning and Restraining the Prey

An important consideration for the *S. fatalis* jaw mechanism is the position of the prey. Biomechanical data show that the *S. fatalis* skull was poorly optimized to resist extrinsic loading from a struggling animal [8], [40], supporting the concept that the prey was restrained prior to the strike, most likely on the ground. The cat’s long, laterally compressed maxillary canines would also have had a high risk of fracturing if used on a moving animal [11], [12], [24], [41]–[43].

**Figure 5:**
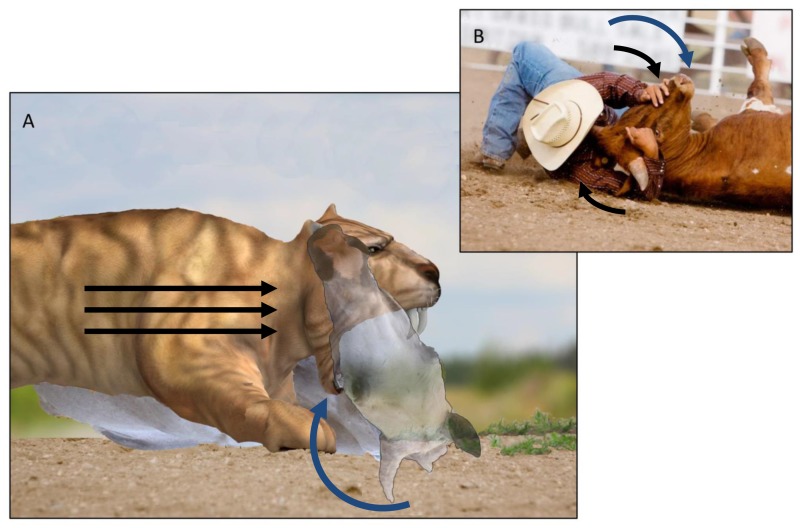
Bulldogging the prey. (A) Using the force of its body to press the buccal aspect of its abducted mandible into the side of the prey’s upturned throat, the cat restrains the prey by locking the prey’s head in a laterally rotated position. This technique is analogous to the rodeo technique of bulldogging a steer (B), in which the cowboy wrestles the steer to the ground by applying a rotational force to the steer’s head (via the muzzle). Note that the cat’s jaws are opened to the maximum gape of 90° and are in position for the strike. Black arrows: forces applied to the head/neck of the prey/steer; blue arrow: direction of rotation of the head of the prey/steer. Credits: (A) Copyright mari_art/Depositphotos, SimpleFoto/Depositphotos and Ralf Juergen Kraft/Shutterstock. (B) Copyright SimpleFoto/Depositphotos.

In the proposed Class 1 Lever Model, the cat restrains the prey on the ground using a variation of the bulldogging model [Fig. 5A] [13], [44]. Wrestling the prey down to its side [39], the cat forces the prey’s head into a laterally rotated position (rotated upward from the ground) ([10]; Fig. 7). The cat then maintains the hold by using the force of its body to press the buccal aspect of its abducted mandible into the side of the prey’s upturned throat. With its head fixed in a lateral position, the prey is unable to rotate its body and return to its feet without first returning its head to a more neutral position. This technique is analogous to a cowboy “bulldogging” a steer (e.g., in the rodeo event of “steer wrestling”) [Fig. 5B], in which the cowboy controls the body of the steer by applying a rotational force to the steer’s head [45]–[47]. However, in contrast to the cowboy, who bulldogs the prey from a standing position and rotates the head by torquing the muzzle [47], the cat in the Class 1 Lever Model bulldogs the prey after pulling it to the ground and controls the prey’s head by torquing the throat. In this manner, the cat can restrain the prey using only its mandible, freeing its forelimbs to power the strike.

### The Class 1 Lever Model: A New Hypothesis for the *S. fatalis* Jaw Mechanism

**Figure 6:**
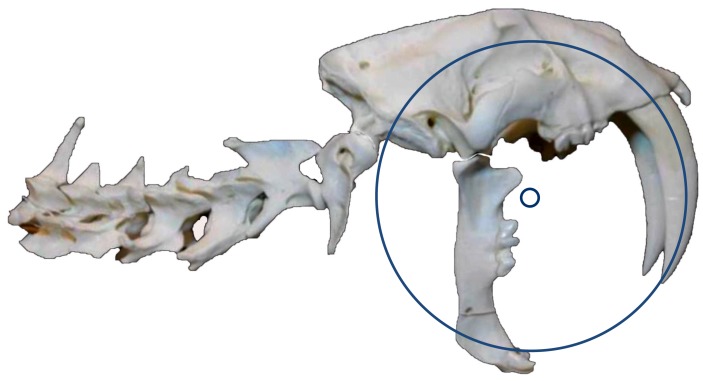
Circular arc of the maxillary canine. The long axis of the maxillary canines can be described by a circle that has its center located slightly anteroventral to the temporomandibular joint (after Wroe et al. [12]). In the Class 1 Lever Model, the use of the center of this circle as a virtual point of rotation would allow the maxillary canines to traverse the prey in the direction of their long axis (i.e., along a circular path).

Because the canine shear-bite cannot explain the force required to augment the anterior rotation of the cranium at the TMJ, the author proposes a new Class 1 Lever Model for *S. fatalis* jaw function. The Class 1 Lever Model is based on the observation that if the mandible is immobilized against the side of the prey, then a dorsally directed force from the extension of the forelimbs can rotate the cranium anteriorly at the TMJ, thereby allowing the forelimb extensors to augment the force of the mandibular adductors when closing the jaws. The model is based on a maximum gape angle of approximately 90° and incorporates a virtual point of rotation located slightly anteroventral to the TMJ (at the center of the maxillary canine curvature) [Fig. 6].

**Figure 7:**
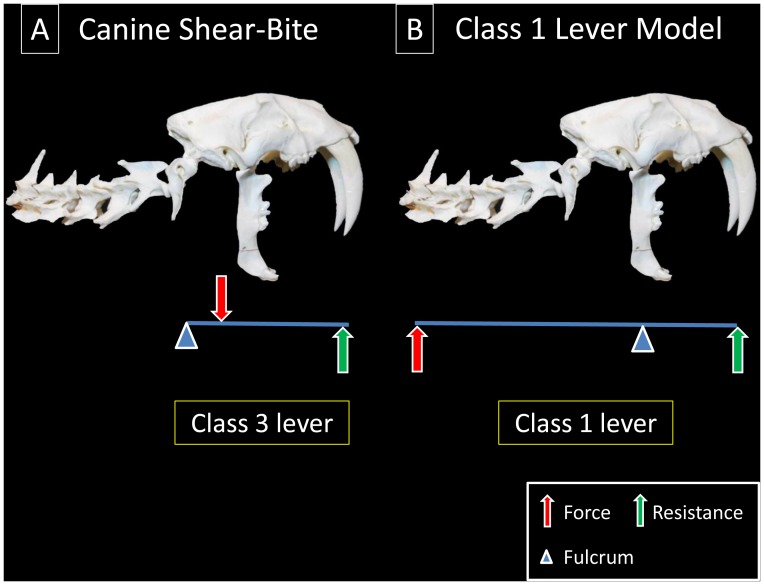
Canine Shear-Bite vs. Class 1 Lever Model. (A) The canine shear-bite is a Class 3 lever with the fulcrum on one side (atlantooccipital joint), the force in the middle (ventral neck flexors) and the resistance on the other side (maxillary canine tips). (B) In contrast, the Class 1 Lever Model is a Class 1 lever: the fulcrum is in the middle (temporomandibular joint), the force is on one side (forelimb extensors), and the resistance is on the other side (maxillary canine tips). Although the canine shear-bite hypothesizes the anterior rotation of the cranium at the temporomandibular joint (enabling the jaws to close), this motion is not compatible with the other aspects of the model.

From a mechanical point of view, the canine shear-bite is a Class 3 lever [Fig. 7A]. The fulcrum is on one side (the AOJ), the force is in the middle (the ventral neck flexors), and the resistance is on the other side (the maxillary canine tips). In contrast, the Class 1 Lever Model is a Class 1 lever [Fig. 7B]. The fulcrum is in the middle (the TMJ), the force is on one side (the forelimb extensors), and the resistance is on the other side (the maxillary canine tips).

There are four hypothesized phases of the *S. fatalis* Class 1 Lever Model: bulldogging, strike, kill and withdrawal.

**Figure 8:**
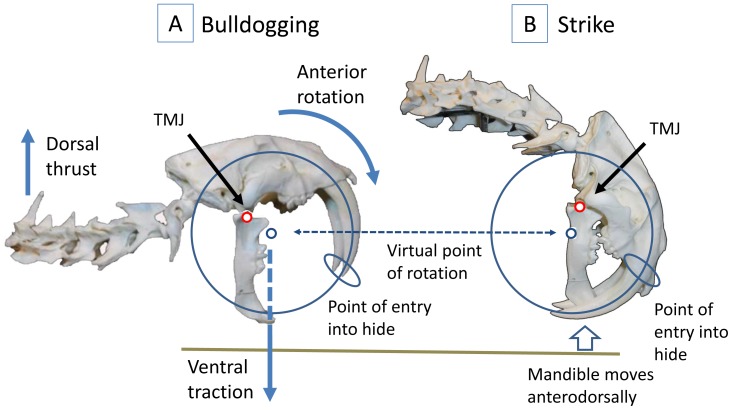
The Class 1 Lever Model. (A) Bulldogging. (B) Strike. The cranium rotates anteriorly at the temporomandibular joint, and the temporomandibular joint rotates anteriorly at the virtual point, enabling the maxillary canines to follow their curvature into the prey. The virtual point in this location may result from a ventrally directed force through this point by the traction of the mandibular canines against the prey (vertical dotted line). Note that the virtual point maintains a constant relationship with the point of entry, whereas the mandible moves relative to the prey as the bite progresses.

**Figure 9:**
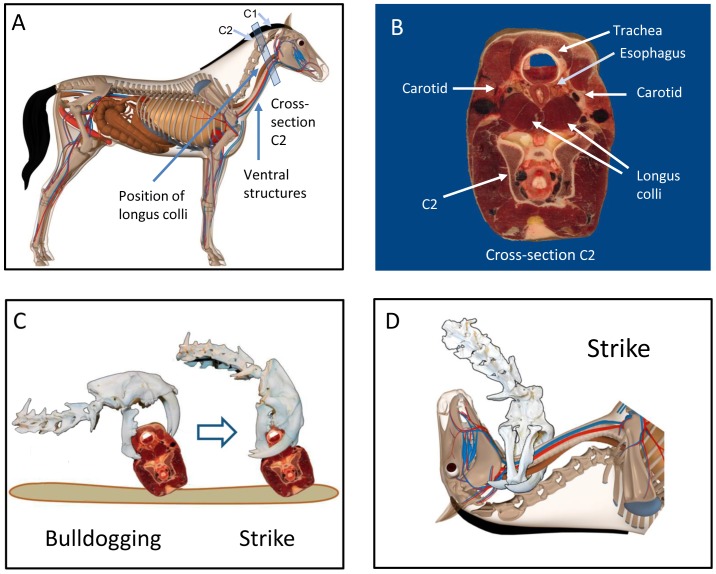
Class 1 Lever Model in action. (A) An illustration of the internal anatomy of the modern horse shows the close proximity and superficial location of the trachea, esophagus and common carotid arteries along the ventral side of the horse’s neck. These structures are separated from the vertebral column by the left and right longus colli muscles. (B) Cross-sectional anatomy of the horse’s neck at the level of C2 with the ventral neck directed upward. The longus colli muscles can be seen positioned between the carotid arteries and the vertebral column. (C, D) Bulldogging and strike positions applied to the prey’s neck. The mandible is positioned on the side of the prey’s throat with the neck of the prey rotated upward from the ground at an angle. Piercing of the maxillary canines through the bilateral longus colli muscles results in circumferential enclosure of the ventral side of the prey’s neck. Credits: (A, D) Internal anatomy of horse. Artist: Friedrich Saurer/Science Source. (B, C) Cross-sectional anatomy of horse’s neck at C2. Copyright: J. Jones, Virginia Polytechnic Institute and State University, reprinted with permission. Accessed at: http://www.vetmed.vt.edu/education/curriculum/vm8644/equineneck/.

1. In the bulldogging phase [Fig. 8A; Fig. S3 in PowerPoint Slide Show S1], the cat’s head and neck are maximally extended; the jaws are opened to the maximum gape of approximately 90°, placing the mandible in a vertical position. With the prey positioned on its side on the ground, the cat presses the buccal aspect of its mandible against the side of the prey’s upturned throat, positioning its mandibular canines/incisors between the dorsal limit of the larynx/trachea and the ventral limit of the vertebral column. Gripping the hide with the mandibular canines/incisors, the cat presses the mandible into the prey, passing the maxillary canines over the ventral surface of the prey’s neck until the tips of the maxillary canines are positioned on the opposite side. As described above (see “Positioning and Restraining the Prey”), this maneuver locks the prey’s head in a laterally rotated position. The body of the cat is in a crouching position; the forelimbs are flexed, and the paws are on the ground. The stabilization of the mandible against the neck of the prey is completed by the prey pushing back against the mandible of the cat, with the mandible and the neck becoming physically immobilized as a result of their mutually counteracting forces.
2. In the strike phase [Fig. 8B; Fig. S4 in PowerPoint Slide Show S1], the cat extends its forelimbs against the ground, elevating its body and creating a dorsally directed force at the caudal aspect of its neck. The cat’s ventral neck flexors stabilize its neck, and the traction of the mandibular canines/incisors against the prey’s hide anchors the mandible to the prey. The cranium rotates anteriorly at the TMJ, driving the maxillary canines into the distal side of the prey’s neck. The simultaneous rotation of the TMJ around the virtual point (the virtual point remains in a constant position, whereas the mandible moves relative to the prey), enables the long axis of the maxillary canines to trace a circular path into the prey. The maxillary canines traverse the full width of the prey’s neck, piercing the bilateral longus colli muscles, and exit the hide on the proximal side adjacent to the mandibular canines [Figs. 9A–D].
3. In the kill phase, the cat’s jaws act to compress the anatomical structures of the ventral side of the prey’s neck (see “Mechanism of Prey Death/Disablement” below).
4. In the withdrawal phase, the cat reverses the order of the strike. The maxillary canines rotate posteriorly, exiting the prey. The mandibular canines/incisors then release the hide.

### Mechanism of Prey Death/Disablement

The Class 1 Lever model is hypothesized to cause the death/disablement of the prey via a compression of the prey’s ventral neck structures. Two different mechanisms are proposed, which may have functioned in concert.

**Figure 10:**
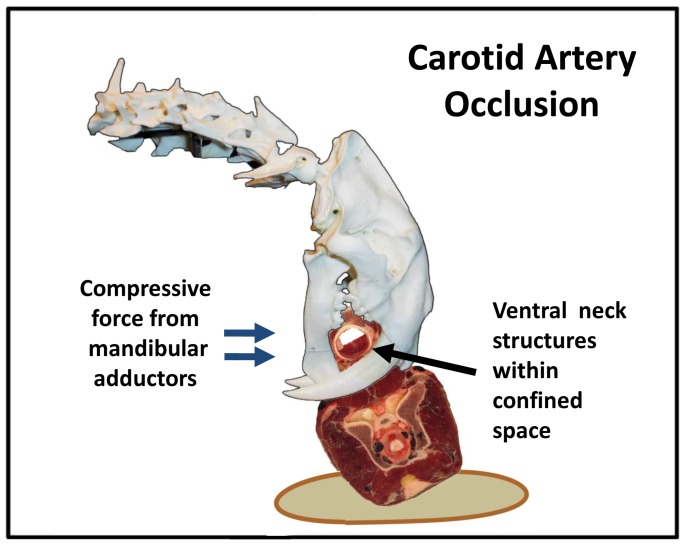
Carotid artery occlusion. The bite of the horse’s ventral neck is bounded on three sides by three rigid structures: the mandible, maxilla and maxillary canines. Closure of the jaws by action of the mandibular adductors (with or without augmentation) circumferentially compresses the tissue in a manner similar to a tourniquet, collapsing the carotid arteries and occluding cerebral blood flow. In the figure, the carotid arteries are not directly visible. Credits: Cross-sectional anatomy of horse’s neck at C2. Copyright: J. Jones, Virginia Polytechnic Institute and State University, reprinted with permission.

In the first mechanism, the passage of the maxillary canines across the full width of the prey’s neck would enable the jaws to form a complete ring around the ventral soft tissue structures, entrapping the prey’s trachea, esophagus and common carotid arteries within a rigid enclosure formed by the cat’s mandible, maxilla and maxillary canines [48], [49]. The cat would then use force from its mandibular adductors (with or without augmentation) to close its jaws in a manner similar to a tourniquet, circumferentially compressing the ventral soft tissue to the point of collapsing the carotid arteries [Fig. 10] (see “Specialized Mechanism for the *S. fatalis* Kill Bite” below).

In the second mechanism, further closure of the cat’s jaws would act to compress the prey’s airway (i.e., larynx/trachea). The compression of the airway would asphyxiate the prey in a manner similar to the suffocating throat bite.

### Evidence in Support of the Class 1 Lever Model

The Class 1 Lever Model is based on the observation that if the mandible is immobilized against the neck of the prey and the cranium rotates anteriorly at the TMJ (enabling the jaws to close), then the most reasonable source to augment the force of the bite is from the extension of the forelimbs, with the neck of the cat functioning as an in-lever. This reasoning suggests that the mandible functions as a hook (to grip the prey) and that the forelimbs are on the ground (to power the bite). Because the forelimbs are on the ground, they would be unable to restrain the prey; therefore, the prey must be restrained using an alternative method. This issue would be resolved by recognizing that when the prey is on its side and its head is rotated laterally, the prey can be restrained by using the mandible alone (via a bulldogging maneuver). The incorporation of a secondary virtual point of rotation for the cranium enables the maxillary canines to penetrate the prey in the direction of their long axis. This model, built by logical extension from the premise of a stationary mandible, makes numerous predictions regarding the functional relevance of different aspects of sabertooth anatomy. An examination of the anatomical features of *S. fatalis* and other sabertooth predators shows broad support for this model [10], [11], [20], [22], [24], [25], [40]–[42], [50].

### The Mandible

**Figure 11:**
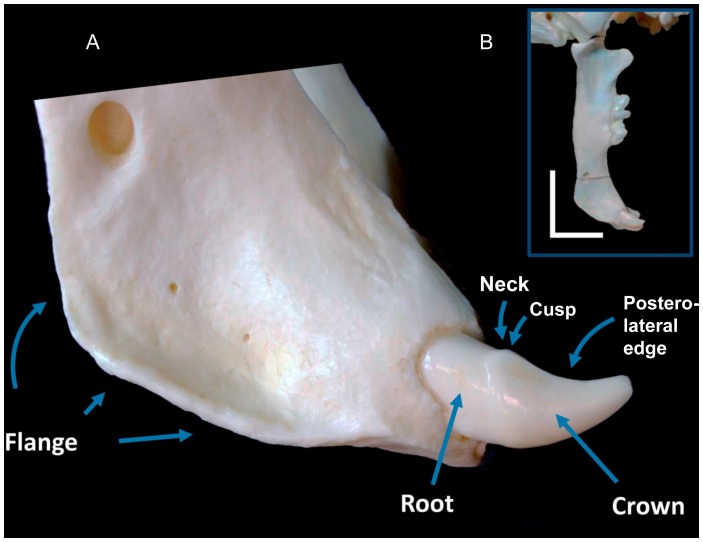
Detailed view of the *S. fatalis* mandible. (A) The crowns of the mandibular canines/incisors have a tapered, crescent-shaped appearance with posteromedial and posterolateral cutting edges. The edges extend to the cervical one-third of the crown where they often appear as small elevated cusps. This morphology suggests that the hide was pierced by the crown before coming to rest on the neck of the tooth, offering a mechanism for mandibular anchoring. Also note the long, thin, ventrolaterally positioned flange. (B) Verticalization of the mandibular symphysis.

The mandible in the Class 1 Lever Model functions to bulldog the prey and grip the hide for the strike [Fig. 11A]. The mandible must withstand tensile forces pulling lengthwise along its body, bending moments applied ventrally against the mandibular canines and perpendicular forces applied against the symphysis. Consistent with these functions, the mandible is particularly robust and made of dense cortical bone [40], and the mandibular canines are strong despite their great reduction in size (relative to the mandibular canines of other large felids) [20], [25]. The ventrolateral mandibular flanges may have helped to resist ventral bending forces as the mandible was pulled dorsally against the hide. Additionally, the verticalization of the anterior surface of the mandibular symphysis [Fig. 11B] (especially when compared with the more gently curved anteroventral surface of the symphysis of conical-toothed felids) [24], [25], [40], along with an increase in symphyseal depth [24], may have helped in resisting bending forces as the mandible was pushed anteriorly into the prey [24], [40], [51]. The use of the mandible to bulldog the prey is also supported by its apparent vertical orientation at the maximum gape [3], [12], [20].

The crowns of the mandibular canines/incisors have a tapered, crescent-shaped appearance with posteromedial and posterolateral cutting edges [4], [20]. The edges extend to the cervical one-third of the crown where they often appear as small elevated cusps, which then angle inward towards the midline to form an elevated cingulum [4], [20]. This morphology suggests that the hide was pierced by the crown [20] before coming to rest on the neck of the tooth, offering a mechanism for mandibular anchoring (the cingulum and cusps may have helped the tooth grip the hide). It should be noted that all sabertooth predators have robust mandibles with similar morphologic features, including the presence of mandibular flanges and verticalization of the mandibular symphysis [11], [14], [40]. The convergent evolution of these common features supports a central role for the mandible in the sabertooth bite mechanism and is not compatible with a ventral rotation of the cranium at the AOJ, which would pull the mandible away from the prey.

### Stabilization of the TMJ

Although the short coronoid process of *S. fatalis* decreases the mechanical advantage of the temporalis muscle in closing the jaws (resulting in small bite forces at high gapes) [3], [8], [12], [13], this arrangement increases the ability of the temporalis to stabilize the TMJ because the force generated by the temporalis at maximum gape is fully directed towards joint compression [19]. This feature is compatible with the Class 1 Lever Model, in which the forelimbs power the bite at high gapes and the temporalis stabilizes the joint.

### Strength of the Forelimbs

In the Class 1 Lever Model, the force contributed by the forelimb extensors augments the force of the bite. Consistent with this hypothesis, a radiographic analysis of the *S. fatalis* humerus has shown thickened cortical bone and an expanded external diameter, indicating an increased ability to withstand compressive and bending forces [42]. Exaggerated forelimb strength appears to be characteristic of all sabertooth predators [50] and is a prominent feature even in primitive taxa such as *Machairodus aphanistus* and *Promegantereon ogygia* [41]. In a study comparing the forelimb strength of sabertooth felids, nimravids and barbourofelidae, the forelimbs were shown to become increasingly robust as the maxillary canines increased in length [50]. Because larger canines would require a greater force to penetrate the hide of the prey, it follows that forelimb strength in sabertooth predators increases with the size of the canines.

### Ventral Neck Flexors

It is generally agreed that *S. fatalis* had powerful neck flexors, as indicated by its well-developed, anteriorly situated mastoid processes [7], [20], [22]. In the canine shear-bite model, these muscles are hypothesized to produce a nodding motion of the head, in which the head flexes ventrally in the direction of the body [20]–[22], [25]. An alternative possibility, also consistent with powerful neck flexors, is a “hunching” of the body ([13]; Fig. 22A), in which the body flexes anterodorsally in the direction of the head. This is the motion employed by the Class 1 Lever Model, in which the immobilization of the mandible against the side of the prey reverses the expected direction of movement. The anterodorsal flexion of the neck in the Class 1 Lever Model may have stabilized the cat’s neck against the dorsally directed force of the forelimbs.

### Maxillary Canines

In the Class 1 Lever Model, the maxillary canines penetrate the prey’s neck and assist in compression of the ventral neck structures. Therefore, rather than cutting perpendicularly to their edges in the manner of sharp-edged knives (i.e., sabers) [10], the primary function of the maxillary canines may have been to pierce through the tissue in a manner similar to semi-circular suturing needles (in surgical suturing, the wrist of the surgeon rotates the needle holder to drive the curved needle into the tissue) [52]–[55]. This piercing function is supported by the teeth’s tapered contours and compressed anterior and posterior edges [20], [56], which may have functioned to concentrate stress and propagate cracks in the prey’s hide [57], [58]. Small serrations along the edges of the teeth [20], [56] may have helped to expand the point of entry as the teeth descended into the tissue [13], [56], [59]. Although the manner of death for the canine shear-bite, in which the maxillary canines cut the carotid, exsanguinating the prey [3], [8], [10], [16], [21], [22], [25], [39], [60], is vivid and intuitively satisfying, it is impractical to expect the dull edges of these teeth [20], [39], [56] to cut through the tough, fracture-resistant hide and connective tissue [39], [61]–[63] of the prey’s neck. The use of the *S. fatalis* maxillary canines in this highly traumatic manner is contradicted by the lack of microwear features found on the teeth [56].

### Course of the Maxillary Canines

With the buccal aspect of the mandible pressed into the side of the prey’s upturned throat, the maxillary canines can traverse the tissue while minimizing the risk of striking bone, thereby protecting the teeth from fracture [10], [11], [13], [43], [56], [64]. Because the tips of the maxillary canines stay within the arc of rotation defined by the mandibular canines [20], as long as the mandibular canines are positioned ventral to the prey’s vertebral column (with the prey’s neck rotated upward from the ground), the maxillary canines will stay ventral to the bone when approaching from the opposite side. Placement of the mandible on the side of the prey’s throat represents a position similar to that of the mandibular throat bite (in both cases, the mandibular and maxillary canines approach the prey’s throat from opposite sides of the prey’s ventral neck) and is consistent with an evolutionary model in which one mechanism transitions into the other (see “Evolution of the Class 1 Lever Model” below).

### Virtual Point of Rotation

The long axis of the *S. fatalis* maxillary canines has a circular curvature with a center that is located slightly anteroventral to the TMJ [6], [12], [13], [20]. In the Class 1 Lever Model, the incorporation of this point as a virtual point of rotation enables the maxillary canines to pierce the tissue in the direction of their long axis, minimizing the tissue resistance and decreasing the strain on the teeth [54], [55]. Although previous authors recognize this point as the center of the maxillary canine curvature, they are unable to explain the rotation at this point within the context of the canine shear-bite [12]. With the mandible placed in a vertical position, the mandibular canines and the virtual point are in approximate vertical alignment (when viewed from a lateral perspective). The ventral pull of the hide against the mandibular canines might help explain the rotation at this point.

### Evolution of the Class 1 Lever Model

The sabertooth complex evolved multiple times in different carnivore lineages [7], [10]–[12], suggesting an important selective advantage for killing prey [3], [10], [20]. Whereas proponents of the canine shear-bite emphasize acute massive blood loss with rapid prey death as the primary selective advantage [3], [8], [10], [16], [21], [22], [25], [39], [60], the Class 1 Lever Model suggests that the initial advantage was an augmentation of the bite force to produce a more powerful suffocating throat bite. An augmentation of the bite force may have been particularly advantageous when subduing prey with thick protective hides [60], [65].

**Figure 12:**
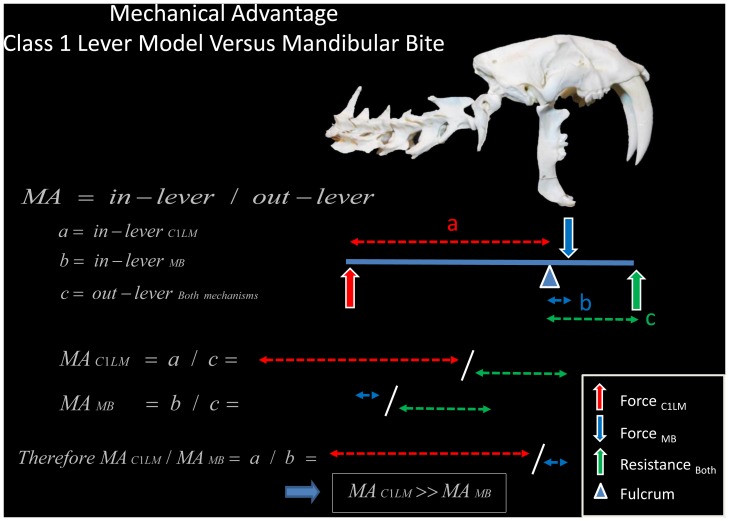
Evolution of the Class 1 Lever Model. The Class 1 Lever Model and mandibular bite share the same fulcrum (the temporomandibular joint) and point of resistance (the canine tips or more posterior teeth) but differ in the position and orientation of the force. The Class 1 Lever Model may have evolved when cats began to use their mandibles to bulldog prey (and thereby restrain it) and then used their forelimbs to augment the bite. The greatly increased mechanical advantage of the Class 1 Lever Model compared to the mandibular bite is visually apparent in the much greater length of its in-lever (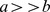). In the above diagram, the mandibular bite has been modeled as a Class 3 lever. Because the cranium and mandible in the mandibular bite move towards one another, the mandibular bite can also be illustrated with the force directed dorsally at the coronoid process and the resistance directed ventrally at the mandibular canine tips. C1LM, Class 1 Lever Model; MB, mandibular bite.

A comparison of the mandibular bite [66] and the Class 1 Lever Model [Fig. 12] shows that the two mechanisms share the same fulcrum and out-lever but differ in the position and orientation of the force. Because the out-lever length is the same, it follows that the difference in MA between the two mechanisms is the difference in length between the two in-levers. The Class 1 Lever Model in-lever (the perpendicular distance from the line of action of the forelimb extensors to the TMJ) is substantially longer than the mandibular bite in-lever (the perpendicular distance from the line of action of the m. temporalis to the TMJ). Therefore, the MA of the Class 1 Lever Model is substantially higher than that of the mandibular bite.

Because the cat’s jaws normally function at a mechanical disadvantage 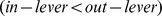 [14], [36], [37], [66] and the bite force is mechanically constrained by its inverse relationship with gape angle [3], [14]–[17], the jaw adductors may be at their physiologic limit for delivering a compressive bite. Bulldogging the prey in the Class 1 Lever Model overrides this constraint by immobilizing the mandible, allowing a dorsally directed force from the extension of the forelimbs to contribute to jaw closure. Because the force contributed by the forelimb extensors is positioned at the caudal aspect of the cat’s neck, the cat’s neck can function as an extended in-lever, increasing the MA and the force of the bite [67].

The blunted, conical-shaped maxillary canines of modern lions [43] do not generally penetrate the hide of large prey [68], and their primary function may be to grip the neck with the compression performed by the more posterior teeth. As a result of the augmented bite force of the Class 1 Lever Model and the stability provided by the restraint of the prey [12], [42], [59], the maxillary canines may have developed a piercing-type function and a more fragile, knife-like morphology [43], [58], [69]. The piercing of the hide by the maxillary canines may have enabled the compressive force of the jaws to be more directly transmitted to the underlying structures.

### Transitional Phase

The observation that the mandibular bite and the Class 1 Lever Model differ primarily in the position and orientation of the force suggests a transitional phase in sabertooth evolution in which the same individual could perform both mechanisms. Approaching from the downed prey's ventral side and clenching the prey's throat in its jaws but unable to collapse the airway (due to the prey’s thick protective hide), the primitive sabertooth uses its mandible to bulldog the prey (and thereby restrain it) before using its forelimbs to augment the bite. This transitional phase for sabertooth evolution is supported by the discoveries of the primitive sabertooth taxa *M. aphanistus*, *P. ogygia* and *Rhizosmilodon fiteae,* in which less fully developed sabertooth features are combined with the more typical craniomandibular morphology of extant cats [3], [10], [24], [29], [41], [70].

Further derivation and elongation of the maxillary canines (requiring proportionally enlarged gapes) may have evolved secondarily to improve the efficacy of the kill bite. In many derived sabertooth taxa such as *Smilodon* and *Barbourofelis*, the incisor arcade may have developed a prehensile function for use in grasping and wrestling the prey [40], [51], [71], with the predator positioning the prey on the ground prior to applying the more fragile maxillary canines.

It should be noted that if the Class 1 Lever Model and mandibular throat bite can be performed by the same individual, then the maximum gape angle (as well as the length of the mandibular/maxillary canines and the canine clearance) would be the same for both mechanisms. A transitional phase for the evolution of the Class 1 Lever Model therefore naturally implies that the mechanism was at least initially related to an augmentation of the bite force and not to an increase in the gape angle. This concept is supported by the observation that the primitive sabertooth taxa *M. aphanistus* [10] and *P. ogygia* [24] had limited adaptations regarding the enlargement of gape. *S. fatalis*, despite its greatly enlarged gape, had a canine clearance similar in size to that of the modern lion [11]. This feature would have limited *S. fatalis* to similarly sized prey [21], although the cat may have concentrated on prey at the upper end of the size range [11]. Dietary studies of bone collagen stable isotope ratios performed at Rancho La Brea show that *S. fatalis* hunted similar prey species as their contemporary conical-toothed counterparts [9], also supporting the concept that the sabertooth bite mechanism was related to a change in killing behavior rather than a change in prey-predator relationships [21].

### Specialized Mechanism for the *S. fatalis* Kill Bite

The highly derived features of the *S. fatalis* cranium and mandible suggest an alternative mechanism for the disablement of the prey. Rather than the force of the bite being primarily absorbed by the rigid structures of the animal’s airway (i.e., the laryngeal and tracheal cartilage), the circumferential enclosure of the ventral side of prey’s neck would enable the jaws to compress the entire soft tissue package. The piercing of the maxillary canines through the full width of the prey’s neck is supported by the extreme length of the teeth as well as the apposition of the mandibular canine tips [20], which would provide an opposing force to the maxillary canines as they pierced outward from the tissue. The complete enclosure of the bite of tissue is supported by the proximity of the lateral edge of the mandible to the lingual surface of the maxillary canine, minimizing the space through which the bite of tissue can protrude. The circumferential compression of the ventral soft tissue would likely initially result in obstruction of the carotid arteries, which would collapse at a pressure of approximately 120 mm Hg (0.016 N/mm^2^), as estimated from the resting coccygeal systolic blood pressure of a normal horse [72]. The physiologic effects of a bilateral carotid artery occlusion is species dependent and is related to the presence or absence of collateral circulation [73]. In humans, the left and right carotid arteries supply approximately 80% of the cerebral blood flow [74]. In experimental rodents, the occlusion of the carotid arteries coupled with or without systemic hypotension is a standard laboratory method for inducing frontal cerebral ischemia [73], [75]. A global reduction in the cerebral blood flow would manifest within seconds as a sudden loss of consciousness (i.e., syncope) [76], [77] that although not immediately fatal for the prey, would promptly disable it. Cessation of blood flow for several more minutes would result in the death of brain tissue [76]; the cat could also collapse the airway of its disabled prey in the manner described for the suffocating throat bite.

### Scope and Limitations of the Present Study

The purpose of the present study was to test the canine shear-bite model in which a ventral rotation of the cranium at the AOJ is used to augment the force of the mandibular adductors. Because the underlying error of the canine shear-bite involves an inconsistency between its different aspects, the emphasis has been placed on functional relationships rather than discrete, quantifiable data. In this study, the author also proposes a Class 1 Lever Model that is supported by anatomical, mechanistic and physiologic considerations. However, the proposed model has not been experimentally tested.

In future studies of the Class 1 Lever Model, emphasis should be placed on a quantitative approach that more precisely defines the model’s mechanical properties. Similar to the methods of experimental paleontology that have been used to study the canine shear-bite [39], the Class 1 Lever Model could be physically tested using the carcasses of extant animals such as bison and horses. Dental wear patterns on fossilized specimens often reflect an animal’s diet and activity and might offer clues regarding its bite mechanics [56]. Additionally, physiologic data from living animals could help elucidate the proposed mechanisms of death.

The mechanistic considerations in the present study suggest that the initial selective advantage of the Class 1 Lever Model involved an augmentation of the bite force at the larger gape angles to more effectively perform the suffocating throat bite. This interpretation leads to a testable hypothesis: the morphologic changes related to bulldogging the prey and performing the bite should precede the appearance of greatly extended maxillary canines and changes related to an increase in gape. This hypothesis finds initial support in the observation that verticalization of the mandibular symphysis [10], [16], [24], [29], [40], [70] and exaggerated forelimb strength [10], [41], [50] are primitive features that persist in all sabertooth predators. In contrast, greatly elongated maxillary canines and morphologic changes related to an increase in gape are features that are primarily found in the more highly derived crown species [3], [10], [11], [16], [28], [51]. A detailed morphometric comparison of a wide range of living and fossil taxa will be required to address this and other issues.

## Conclusion

The experiments in the present study show that the *S. fatalis* canine shear-bite, as originally proposed by Akersten in 1985 and widely accepted by the paleontology community, is mechanically impossible. Contrary to Akersten’s hypothesis, the head-depressing musculature (i.e., the ventral neck flexors inserting along the mastoid process and rotating the cranium ventrally at the atlantooccipital joint) would not function to close the jaws but would instead rotate the cranium and the mandible ventrally together. Although the canine shear-bite is often illustrated with the mandible immobilized and the cranium rotated anteriorly at the temporomandibular joint (which would function to close the jaws), this motion is not explained by Akersten’s model.

As a result, this paper proposes a new Class 1 Lever Model for *S. fatalis* jaw function. The mandibular canines function as a hook, anchoring the mandible to the prey, and the forelimbs provide the force, rotating the head anteriorly at the temporomandibular joint. The maxillary canines penetrate the full width of the prey’s neck, aided by the closely opposed mandibular canines, and the compression of the structures of the prey’s ventral neck acts to suffocate the prey and/or occlude the cerebral blood flow. The model incorporates a virtual point of rotation as previously described in the paleontology literature and utilizes a maximum gape angle of approximately 90°. The model also incorporates a bulldogging maneuver, in which the prey is restrained by the cat’s mandible alone. The Class 1 Lever Model is mechanically possible and may have potentially evolved from the mandibular throat bite. Although further analysis and experimentation are required to clarify the mechanistic details, the Class 1 Lever Model should be considered a viable alternative to the currently accepted canine shear-bite.

## Supporting Information

PowerPoint Slide Show S1 **Jaw Function in Smilodon fatalis: Canine Shear-Bite Versus Class 1 Lever Model. Figure S1, Mandibular Phase.** With the prey on its side, the cat positions its gaping mouth against the prey’s ventral neck and uses its mandibular adductors to press its jaws into the prey. The prey is restrained by the cat’s forelimbs (not illustrated). **Figure S2, Neck-powered Phase.** The ventral neck flexors rotate the cranium ventrally at the atlantooccipital joint, driving the maxillary canines into the prey. Note that the mandible is pulled away from the prey while the jaws remain open. **Figure S3, Bulldogging.** With the prey positioned on its side, the cat bulldogs the prey by pressing the buccal aspect of its abducted mandible into the side of the prey’s upturned throat, locking the prey’s head in a laterally rotated position. **Figure S4, Strike.** With its mandible immobilized against the neck of the prey, the cat uses a dorsally directed force from the extension of its forelimbs to rotate the cranium anteriorly at the temporomandibular joint, thereby augmenting the force of its mandibular adductors when closing its jaws.
(PPT)
